# The Spread of Multi Drug Resistant Infections Is Leading to an Increase in the Empirical Antibiotic Treatment Failure in Cirrhosis: A Prospective Survey

**DOI:** 10.1371/journal.pone.0127448

**Published:** 2015-05-21

**Authors:** Manuela Merli, Cristina Lucidi, Vincenza Di Gregorio, Marco Falcone, Valerio Giannelli, Barbara Lattanzi, Michela Giusto, Giancarlo Ceccarelli, Alessio Farcomeni, Oliviero Riggio, Mario Venditti

**Affiliations:** 1 Gastroenterology, Department of Clinical Medicine, “Sapienza” University of Rome, Rome, Italy; 2 Department of Infectious disease, “Sapienza” University of Rome, Rome, Italy; 3 Department of Public Health and Infectious Diseases, Statistics Section, “Sapienza” University of Rome, Rome, Italy; University Medical Center Groningen, NETHERLANDS

## Abstract

**Background:**

The spread of multi-resistant infections represents a continuously growing problem in cirrhosis, particularly in patients in contact with the healthcare environment.

**Aim:**

Our prospective study aimed to analyze epidemiology, prevalence and risk factors of multi-resistant infections, as well as the rate of failure of empirical antibiotic therapy in cirrhotic patients.

**Methods:**

All consecutive cirrhotic patients hospitalized between 2008 and 2013 with a microbiologically-documented infection (MDI) were enrolled. Infections were classified as Community-Acquired (CA), Hospital-Acquired (HA) and Healthcare-Associated (HCA). Bacteria were classified as Multidrug-Resistant (MDR) if resistant to at least three antimicrobial classes, Extensively-Drug-Resistant (XDR) if only sensitive to one/two classes and Pandrug-Resistant (PDR) if resistant to all classes.

**Results:**

One-hundred-twenty-four infections (15% CA, 52% HA, 33% HCA) were observed in 111 patients. Urinary tract infections, pneumonia and spontaneous bacterial peritonitis were the more frequent. Forty-seven percent of infections were caused by Gram-negative bacteria. Fifty-one percent of the isolates were multi-resistant to antibiotic therapy (76% MDR, 21% XDR, 3% PDR): the use of antibiotic prophylaxis (OR = 8.4; 95%CI = 1.03-76; P = 0,05) and current/recent contact with the healthcare-system (OR = 3.7; 95%CI = 1.05-13; P = 0.04) were selected as independent predictors. The failure of the empirical antibiotic therapy was progressively more frequent according to the degree of resistance. The therapy was inappropriate in the majority of HA and HCA infections.

**Conclusions:**

Multi-resistant infections are increasing in hospitalized cirrhotic patients. A better knowledge of the epidemiological characteristics is important to improve the efficacy of empirical antibiotic therapy. The use of preventive measures aimed at reducing the spread of multi-resistant bacteria is also essential.

## Introduction

Antimicrobial resistance represents a general public health concern, although the size and the characteristics of the problem may vary geographically, temporally and according to the healthcare setting [1, 2]. Infections caused by resistant pathogens are usually encountered in the hospital setting, besides, the diffusion of health-care assistance beyond the hospital is currently leading to a spread of antibiotic resistant pathogens in the general healthcare system. This problem is associated with worse patients’ clinical outcomes during infections.

Cirrhotic patients, in their advanced stage, are highly susceptible to infections, are frequently in need of assistance from the health-care system and of antibiotic therapies (either for treatment or for prophylaxis) and are subject to recurrent hospitalizations for complications of the disease.

Several authors have recently warned for an increasing incidence of infections caused by multi-resistant bacteria in cirrhosis [3–5].

A well-accepted definition of multi- resistant organisms is based on the resistance in vitro to at least one agent in three or more antimicrobial categories [6, 7]. Although the names of certain multi-resistant organism describe resistance to only one agent (e.g., methicillin-resistant *Staphylococcus aureus* and vancomycin-resistant enterococci), these pathogens are frequently resistant to most available antimicrobial agents. For epidemiological purposes, due to the large variety of the pathogens included in this group, a further sub-classification has been proposed in recent years: pathogens non-susceptible to at least one agent in all but two or fewer antimicrobial categories (i.e. bacterial isolates remain susceptible to only one or two categories) are classifiable as Extensively-drug Resistant (XDR) and those nonsusceptible to all agents in all antimicrobial categories as Pan-drug Resistant (PDR) [8].

In the last years, epidemiology, risk factors and clinical outcomes of multi-resistant infections, particularly according to their epidemiological origin, have raised special attention in cirrhosis. The above proposed classification could be highly relevant for epidemiological purposes as it introduces a harmonized terminology, takes into consideration the severity of bacterial resistance and therapeutic consequences.

To the best of our knowledge, we applied the classification in MDR, XDR and PDR in cirrhotic patients for the first time, describing a high rate of MDR and XDR infections [9]. A recent retrospective study limited to bloodstream infections, also reported a high rate of Gram negative MDR and XDR pathogens among cirrhotic patients [10].

Our study was carried out prospectively in order to assess characteristics, risk factors and outcome of multi- resistant infections in a large series of hospitalized cirrhotic patients. For this aim we considered only patients with microbiologically documented infections (MDI). Episodes of infection were classified according to the different degree of multi-resistance (MDR, XDR, PDR). The efficacy of currently recommended empirical antibiotic therapy was also evaluated.

## Patients and Methods

### Patients

All cirrhotic patients consecutively admitted to our Department University hospital from October 2008 to June 2013, were considered for enrollment. Only patients admitted with MDI or developing it during hospitalization were included in the study.

Concomitant immunosuppressant conditions (HIV infection, high dose corticosteroid treatment and other immunosuppressive therapies) and hepatocellular carcinoma out of the Milan criteria were considered as exclusion criteria.

At admission, relevant baseline demographic, clinical and biochemical data were recorded. The data collection was performed taking carefully into account the possible risk factors for infections caused by multi-resistant bacteria (site of acquisition of the infection, recent use of antibiotics, previous hospitalization, previous infections, invasive procedures, long-term quinolone-prophylaxis). The severity of liver disease was assessed using the Child–Pugh and the model of end-stage liver disease (MELD) scores.

The study was approved by the local Ethical Committee Review Board (Policlinico Umberto I) and a written informed consent was obtained by all the participants to allow the collection of their individual data pertaining their previous history and hospitalization.

### Assessment of infections and antibiotic treatments

Episodes of infections were always actively sought out either at admission or during the hospital stay.

A detailed form concerning the infectious episodes, was compiled including the epidemiological characteristics, the site of infection, the result of positive microbiology cultures, the results of white blood cells count and inflammatory indices, the antibiotic therapy. Information regarding the clinical events during hospitalization were also collected (i.e., efficacy of the initial empirical antibiotic therapy, complications related to infection, resolution, and hospital mortality).

The diagnosis of bacterial infection was based on previously reported standard criteria [3].

According to their epidemiological characteristics, infections were classified as follows: (1) Hospital acquired (HA) in case of infection onset at least 48 hours after the admission, or within 10 days of leaving the hospital, or if the patient was coming from another department. (2) Health-care associated (HCA) if the diagnosis was made at hospital admission or within 48 hours of hospitalization in patients with any of the following criteria: a) had attended a hospital or a hemodialysis clinic or received intravenous chemotherapy in the last 30 days, b) was hospitalized for at least two days or had undergone surgery during the 90 days before infection or c) had resided in a nursing home or a long-term care facility. (3) Community acquired (CA) if the diagnosis of infection was made within 48 hours of hospitalization and the patient did not fulfil the criteria for HCA infection (had no recent contact with the health-care system) [11, 12].

### Prophylaxis and treatment of infections

Patients with a history of gastrointestinal bleeding or previous spontaneous bacterial peritonitis (SBP) or subjected to invasive procedures for which prophylaxis was indicated, were treated with antibiotic therapy according to the general guidelines or our hospital protocol for invasive procedures.

When an infection was suspected, after a prompt collection of the culture specimens, patients were immediately treated with broad-spectrum antibiotics according to the likely site of infection, in accordance with current guidelines [13] and considering the local epidemiology. Antimicrobial treatment before the result of cultures and the tests of the in vitro susceptibility was considered empirical, and became definite only when this information was available. Empirical antibiotic treatment was considered appropriate only when the isolated bacteria was found to have in vitro susceptibility to that antibiotic. If not the antibiotic therapy was accordingly changed.

### Classification of multi-resistance

We considered “multi-resistant” all pathogens resistant to three or more antimicrobial classes.

Among them, according to the international classification of the different degrees of multi-resistance, infections were divided as follows [8]: (a) "Multidrug-resistant" (expressed as MDR) if the pathogen was resistant to three or more antimicrobial classes, but susceptible to at least three classes. (b) "Extensively Drug-Resistant" (XDR) when the pathogen was sensitive only to one or two classes. (c) "Pandrug-Resistant" (PDR) when the pathogen was resistant to all antimicrobial classes.

For these definitions, bacterial isolates were considered resistant to an antimicrobial class when they were not susceptible to at least one member of the class.

### Statistical analysis

All the values are reported as means ± SD and p values <0,05 were considered statistically significant.

Differences among CA/HA/HCA groups were compared using the chi-squared test or ANOVA. Rejection of the null hypothesis implies that at least one of the three groups was different from the other two. When the hypothesis was significant, we performed post-hoc multiplicity adjusted multiple comparison.

Differences between MDR and XDR groups were evaluated by means of the Student’s test for unpaired data or the chi-squared test, as appropriate. The paired sample t-test was used to longitudinally compare psychometric performances. The correlation between psychometric tests and inflammatory indices was analyzed by Pearson’s correlation Logistic regression analysis was employed to identify possible predictors of the cognitive impairment. We do not further adjust for multiple comparison with respect to the outcomes considered and report raw p-values, as any adjustment would lead to the same conclusions regarding statistical significance. The software used for the analysis was NCSS (Number Cruncher Statystical System) 2007.

## Results

### Patients

During the enrollment period, 424 cirrhotic patients, were hospitalized in our ward. Twenty-five patients were excluded: 5 because of the concomitant use of immunosuppressive medications and 20 because of a diagnosis of hepatocellular carcinoma out of the Milan criteria. A bacterial infection was documented at admission or during hospitalization in 173 (39%) patients. Sixty-two patients had a culture-negative infection, 111 had a diagnosis of MDI.

Demographic characteristics and severity of liver disease were similar in patients with MDI and in those with culture negative infections (data not shown).

The median age of the 111 patients was 60 ± 13 years, 64% were males. Origin of liver disease was hepatitis C in 42%, hepatitis B in 10%, alcohol abuse in 23% (12% of the patients were active alcohol abusers at the time of admission). The majority of patients had a decompensated liver disease (53% Child-Pugh B, 36% Child-Pugh C) and the mean MELD score was 16.5 ± 7.

The main reasons for admission were the onset of portal-hypertension related complications (ascites 23.6%, hepatic encephalopathy 26.4%, acute gastrointestinal bleeding 4.5%), elective invasive procedures (10.9%), fever (7.3%) or indirect signs of infection (6.4%). At admission, 68 patients had evidence of ascites, 73 patients had esophageal varices (in 29% large varices at risk of bleeding). Fifty-nine patients had hepatic encephalopathy either at admission or during hospitalization. Twenty-one patients had a diagnosis of hepatocellular carcinoma within the Milan criteria. Forty-one patients (37%) had a diagnosis of diabetes.

#### Patients characteristic according to the epidemiology of the infections

The demographic, clinical and biochemical data of the patients enrolled in the study according to epidemiological classifications of the infections are summarized in Table 1.

**Table 1:** Demographic, clinical and biochemical characteristics of the 111 patients enrolled in the study according to the epidemiological class of the first infection: Community Acquired (CA), Hospital Acquired (HA) and Healthcare Associated (HCA).

	CAI (18)	HAI (51)	HCAI(42)	P (*)
Males, n (%)	11 (61)	35 (69)	24 (58)	n.s.
Age (years)	60.7 ± 19	58.4 ± 14	62.2 ±10	n.s.
**Main etiology of the liver disease, n (%)**				n.s.
HBV	2 (11)	5 (10)	2 (5)	
HCV	7 (39)	19 (37)	18 (44)	
HBV and HCV	1 (5.5)	2 (4)	1 (2.5)	
Alcohol	4 (22)	10 (20)	11 (27)	
Others	4 (22)	15 (29)	9 (21.5)	
**Main cause of admission, n (%)**				n.s.
Ascites	5 (28)	12 (23.5)	9 (22)	
Hepatic encephalopathy	6 (33)	8 (16)	15 (37)	
Variceal bleeding	0 (0)	5 (10)	0 (0)	
Suspected infections	2 (11)	6 (12)	7 (17)	
Elective procedures	1 (6)	7 (14)	4 (10)	
Other	4 (22)	13 (25.5)	6 (15)	
Child-Pugh class C, n (%)	2 (11)	21 (41)	17 (41)	n.s.
MELD score	14.8 ± 8.5	17.5 ± 8.1	15.7 ± 4.5	n.s.
Serum bilirubin (mg/dL)	5.7 ± 12.5	10 ± 17.4	4.8 ± 4.6	n.s.
Serum albumin (mg/dL)	3.1 ± 0.6	3.1 ± 0.8	3.5 ± 4.1	n.s.
International Normalized Ratio	1.5 ± 0.4	1.6 ± 0.5	2 ± 2.1	n.s.
Platelets (num/mm3)	128330 ± 86500	118090 ± 128490	141330 ± 130530	n.s.
Serum creatinine(mg/dl)	1.3 ± 1.2	1.2 ± 1	1.2 ± 1.2	n.s.
Blood Urea Nitrogen (mg/dL)	35 ± 29	44 ± 36	39.5 ± 25	n.s.
Serum sodium (mmol/L)	135.7 ± 4.9	134.7 ± 5.7	130 ± 21	n.s.
Hepatocellular carcinoma, n (%)	3 (17)	12 (23.5)	6 (14.5)	n.s.
Ascites, n (%)	12 (67)	33 (65)	22 (53.5)	n.s.
Hepatic encephalopathy, n (%)	8 (44.5)	25 (49)	26 (63.5)	n.s.
Sepsis, n (%)	5 (28)	29 (57)	11 (27)	0.01
Temperature >37,5°C, n (%)	5 (28)	13 (25.5)	10 (24.5)	n.s.
Heart Rate (beats/min)	82.5 ± 11	74 ± 12	78 ± 13	0.04
Respiratory rate (breath/min)	17.8 ± 4.6	16.7 ± 2	15.9 ± 2	0.04
White Blood Cells (cell/mm3)	6105 ± 3250	5820 ± 3643	7660 ± 4350	0.08
Sedimentation rate (mm/h)	38 ± 24	26.5 ± 23	35 ± 29	n.s.
C- reactive protein	3 ± 3.7	5.5 ± 10.3	2.7 ± 2.5	n.s.

(*) p-values for testing at least one significant difference among the three groups.

The patients in the three groups did not differ for age, sex, etiology of cirrhosis, reasons for hospitalization, diagnosis of hepatocellular carcinoma and active alcohol abuse. Severity of liver disease, as evidenced by the MELD and Child-Pugh score were also similar. HA infections were more frequently associated with sepsis. In-hospital mortality, although higher in patients with HA and HCA infections, was not significantly different from CA.

### Microbiologically documented infections in the different epidemiological classes

Apart from the 111 diagnosis of MDI during admission, among the enrolled populations, 13 patients experienced a second MDI during the same hospitalization. Therefore, we considered 124 episodes of infection in 111 patients.

Characteristics of the 124 MDI according to the epidemiological classes are reported in Table 2.

**Table 2:** Characteristics of the 124 episodes of infections according to the epidemiological class (community-acquired (CA), hospital acquired (HA), healthcare associated (HCA) infections).

	CA (18)	HA (64)	HCA (42)	P (*)
Urinary tract infections, n (%)	12 (67)	33 (51)	31 (75.5)	0.03
Pneumonia, n (%)	1 (5.5)	10 (16)	4 (7)	n.s.
Spontaneous bacterial peritonitis, n (%)	3 (17)	4 (6.5)	3 (7)	n.s.
Spontaneous bacteraemia, n (%)	1 (5.5)	7 (11)	0 (0)	n.s.
Biliary tract infections, n (%)	0 (0)	2 (3)	0 (0)	n.s.
Other infections, (Skin, gastrointestinal, lymphagitis, bursitis), n (%)	1 (5.5)	8 (13)	4 (10)	n.s.
Gram positive/ negative// Mixed, n (%)	5 (28)/ 13 (72)/ 0 (0)	38 (59)/ 22 (35)/ 4 (6)	15 (37)/ 23 (54)/4 (10)	n.s.
Enterobacteriaceae	13 (72)	20 (32)	22 (51)	0.005
*E*. *coli*	12 (67)	14 (22)	16 (37)	0.001
*K*. *pneumoniae*	1 (6)	5 (8)	3 (7)	n.s.
*P*. *mirabilis*	0 (0)	1 (2)	3 (7)	n.s.
*Enterococcus*	2 (11)	14 (21)	9 (22)	n.s.
*Enterococcus* +Enterobacteriaceae	0 (0)	4 (6.5)	3 (7)	n.s.
S. aureus	2 (11)	12 (19)	1 (2.5)	0.04
Coagulase neg staphylococcus	1 (5.5)	3 (5)	3 (7)	n.s.
Other	0 (0)	11 (17.5)	4 (10)	n.s.
Multi-resistant infections, n (%)	4 (22)	34 (53)	26 (62)	0.008

(*) p-values for testing at least one significant difference among the three groups.

Urinary tract infections (UTIs), followed by pneumonia and SBP, were the most frequent infections in all the epidemiological classes.

Gram negative bacteria tended to be more frequent in CA and HCA infections while Gram-positive ones in HA. In the latter group, the isolation of Gram-positive bacteria was associated with a higher number of invasive procedures during hospitalization (2.6 ± 1.7 vs 1.9 ± 1.9, p = 0.05).

Enterobacteriaceae (44.3%) (particularly *Escherichia coli*, *Klebsiella pneumoniae* and *Proteus mirabilis*), Enterococcae (*Enterococcus faecium* and *Enterococcus faecalis*) (19.7%), *Staphilococcus aureus* (12.3%) and coagulase-negative Staphylococci (5.7%) were the pathogens most frequently responsible for infection.

#### Multi-resistant infections

Multi-resistant bacteria were identified in half of healthcare-related infections (HCA and HA; Table 2).

Spontaneous bacteremia, SBP and pneumonia were the infections with the higher probability of multiresistant bacteria (87%, 60% and 57%, respectively). At variance, UTIs were more frequently caused by organisms sensitive to multiple antibiotic classes (57%).

Concerning the susceptibility of the isolated pathogens, *Klebsiella pneumoniae* and *Proteus mirabilis* were multi-resistant in almost all the cases (82% and 100%, respectively), *Enterococcae* and *Staphylococcus aureus* in about half of the cases (59% and 53%, respectively). *Escherichia coli* and coagulase-negative Staphylococci were multi-resistant in 38% and 29%, respectively.

As shown in Table 3, the current or previous contact with the healthcare environment (HA or HCA infections) (p = 0.02), the use of antibiotics in the last month (p = 0.04) and the chronic antibiotic prophylaxis (p = 0.01) were factors associated with multi-resistant infections at univariate analysis.

Healthcare associated origin of the infection (HA and HCA) (OR 3.7; 95% CI 1.05–13; P = 0.04) and the use of antibiotic prophylaxis (OR 8.4; 95% CI 1.03–76; P = 0.05) were selected as independent predictors for the development of bacterial multi-resistant infections at multivariate analysis.

**Table 3:** Possible risk factors and outcomes of multi-resistant and non multi-resistant infections.

	Multi-resistant infections (64)	Non Multi-resistant infections (60)	P
CAI/ HAI/ HCAI	4 (6.5)/ 34 (53)/ 26 (40)	14 (23)/ 30 (50)/ 16 (27)	0.02
Use of antibiotics in the last 30 days, n (%)	24 (39)	13 (22)	0.04
Antibiotic prophylaxis, n (%)	9 (14.5)	1 (2)	0.01
Hospitalizations in the last 6 months, n (%)	40 (64.5)	38 (63)	n.s.
Infections i the last 12 months, n (%)	25 (40)	25 (42)	n.s.
Failure of empirical antibiotic therapy, n (%)	37 (58)	22 (37)	0.004
Deterioration of Child-Pugh score, n (%)	34 (55)	18 (35)	0.005
Deterioration of MELD score, n (%)	34 (55)	21 (35)	0.01
Deterioration in renal function, n (%)	11 (17)	8 (13)	n.s.
In-hospital mortality, n (%)	19 (30)	11 (18)	n.s
Hospital stay (from the diagnosis of infection) (days)	20.2 ± 21.6	17.8 ± 13	n.s.

#### Epidemiology of various degrees of multi resistance

Applying the classification of multi-degree of resistance, we observed 47 MDR, 15 XDR and 2 PDR.

By a time-dependent analysis, we observed, over time, a trend toward an increase in the isolation of XDR/PDR pathogens. In particular, the number of XDR/PDR isolated bacteria were almost doubled in the last two years (16% in 2008–2009 and 20% in 2010–2011 and 36% in 2012–2013).

Among the episodes due to multi-resistant pathogens, 57% of spontaneous bacteremia, 27% of SBP, 25% of pneumonia and 15% of UTIs were re-classified as XDR.

Concerning the susceptibility of the main specific isolated pathogens, among the multi-resistant ones, *Staphylococcus aureus*, *Enterobacteriaceae*, *Enterococcae* were re-classified as XDR respectively in 37.5%, 27% and 11%.

No specific risk factors for the severity of the degree of multi-resistance could be found in our cohort (Table 4).

**Table 4:** Possible risk factors and outcomes of Multi-Drug Resistant (MDR) and Extensively- Drug Resistant (XDR) infections (Pan-drug Resistant infections were not considered due to the small sample size).

	MDR (47)	XDR (15)	P
CAI/ HAI/ HCAI, n (%)	2 (4)/ 26 (55)/ 19 (40.5)	2 (13)/ 7 (47)/ 6 (40)	n.s.
Antibiotic prophylaxis, n (%)	9 (19)	0 (0)	n.s.
Additional bed, n (%)	20 (42.5)	10 (67)	n.s.
Use of antibiotics in the last 30 days, n (%)	18 (38)	6 (40)	n.s.
Hospitalizations in the last 6 months, n (%)	33 (70)	7 (47)	n.s.
Infections in the last 12 months, n (%)	19 (40,5)	6 (40)	n.s.
Failure of empirical antibiotic therapy, n (%)	28 (60)	14 (93)	0.01
Deterioration in renal function, n (%)	8 (17)	3 (20)	n.s.
In-hospital mortality, n (%)	12 (25)	6 (40)	n.s.
Hospital stay (from the diagnosis of infection) (days)	17.8 ± 12.7	28 ± 37.8	n.s.

#### Empirical antibiotic failure and outcomes in multi-resistant infections

As expected, a higher rate of failure of empirical antibiotic therapy was observed in patients with MDR (60%) and in XDR infections (90%) compared to patients with an infection due to a non multi-resistant pathogen (37%) (Tables 3 and 4).

A deterioration of liver function was documented in patients with multi-resistant infections. There was a trend to a higher occurrence of deterioration of renal function, to a longer hospital stay (time between diagnosis of infection and discharge) and to a higher mortality rate, although without reaching statistical significance, during hospitalization in the multi-resistant group compared to non multi-resistant and in XDR group vs MDR (Tables 3 and 4).

Specifically, we observed a significantly higher rate of deterioration of renal function (23% vs 7%; p = 0.02), a longer hospital stay (20.6 ± 14 vs 13.6 ± 11; p = 0.003) and a higher in-hospital mortality (35% vs 10%, p = 0.001) in case of failure of the empirical antibiotic treatment.

## Discussion

While a significant improvement in the clinical management of cirrhotic patients has been seen in recent years, bacterial infections are still very frequent and cause severe complication in this group of patients [3].

In cirrhotic patients, the infections caused by multi-resistant pathogens represent a growing problem, even more than in the general population. In the last decade, several studies have addressed the epidemiological characteristics of infections and the prevalence of antibiotic resistances in patients with cirrhosis [3–5, 10, 14]. However, the microbiological characteristics may largely vary according to time and place, so the best way to drive the empirical antibiotic therapy is to known the actual local epidemiology.

Our single centre prospective study, was aimed to assess epidemiological characteristics, risk factors and outcomes of multi-resistant infections in hospitalized cirrhotic patients.

For these aims, we considered all infectious sites and not only bloodstream infections or SBP, as in previous studies [4, 10]: UTIs and pneumonia were the prevalent infections in our cohort. UTIs are the most frequent type of infection in the majority of the clinical records, representing one of the main reason for administering empirical antibiotic therapy. Therefore, we think that the inclusion of this kind of infection enhances the clinical strength of our study.

Concerning the microbiological characteristics of our cohort, *Enterobacteriaceae*, followed by *Enterococcae*, were the pathogens most often responsible for infections. At the same time, as previously described, a growing prevalence of non-enterococcal Gram-positive bacteria (*Staphylococcus aureus* and coagulase-negative Staphylococci) was observed, particularly in the setting of HA infections and especially in patients undergone to multiple invasive procedures [5, 15]. A multi-resistant pathogen was isolated in about half of patients. This percentage is much higher than that reported by the majority of studies conducted in other countries [4, 5, 15, 16], due to the higher prevalence of multi-drug resistance in our hospital and to the epidemiological characteristics of the enrolled patients (high rate of HCA and HA infections). As we previously reported, the majority of multi-resistant infections occurred, in fact, in patients hospitalized or with a recent contact with the hospital environment (53% in HA, 62% in HCA vs 22% in CA).

A current or previous contact with the healthcare environment and the use of an antibiotic prophylaxis were both selected among the independent risk factors for multi-resistant bacterial infections suggesting a relevant role for the antibiotic pressure present in both this conditions.

Applying the international classification proposed in general population [8] to further stratify the multiresistant infections, we documented that a relevant amount of multi-resistant infections were XDR (when the pathogen was sensitive only to one or two classes) and that this percentage was doubling in the last years as shown by a time-dependent analysis. No specific risk factors for the severity of the degree of multi-resistance could be found in our cohort, probably due to the small size of this group.

As shown in our study, the increase in the prevalence of MDR and XDR infections among cirrhotic patients is associated to a more frequent failure of empirical antibiotic therapy. A deterioration of liver function was documented in patients with multi-resistant infections. A higher morbidity and mortality was observed progressively in patients with MDR and XDR, particularly in case of failure of the empirical antibiotic treatment.

In summary, although the results of the current study, deriving from a single centre experience with a specific microbiological pattern, cannot be generalized, our study may provide several practical messages.

First, the high incidence of infections related to the healthcare system (HA and HCA) and their close relationship with multi-resistance underline the need to improve preventive measures against bacterial infections in hospital setting. A relocation of care from hospital to the home assistance and a better use of isolation precautions during hospitalization may be essential to limit the spread of multi-resistant organisms. Hygienic measures, the use of catheters (both urinary and vascular) only when strictly necessary and the removal of these devices as soon as possible, may be the first steps. Furthermore, the indications for antibiotic prophylaxis should be carefully evaluated accordingly to the actual context. Second, the knowledge of the own epidemiology is extremely important considering the high clinical relevance of a correct empirical antibiotic therapy. In this scenario, a better stratification of the multi resistances may allow to better characterized this growing problem. Moreover, the high rate of first line antibiotic treatment failure in HCA, would imply the need for second-line therapies as well as in HA, although randomized trial are needed.

Third, as emphasized in the general population [17], the risk of a rapid increase in pathogens potentially resistant to every drug on the market is increasing due to a “delay” in the development of new antimicrobial classes. Anyway, in our study, although the number of XDR pathogens was relevant, the rate of PDR was still low leaving a little space to apply preventive measures.

An additional factor of improvement, finally, can be derived from the acquisition of the technology (now already available in several centers) needed to speed up bacterial isolation to quickly use of targeted antibiotic therapy.
